# Inoculation of raccoons with a wild-type-based recombinant canine distemper virus results in viremia, lymphopenia, fever, and widespread histological lesions

**DOI:** 10.1128/msphere.00144-23

**Published:** 2023-06-14

**Authors:** Dagmar Roelofs, Katharina S. Schmitz, Geert van Amerongen, Laurine C. Rijsbergen, Brigitta M. Laksono, Anouskha D. Comvalius, Sham Nambulli, Linda J. Rennick, Peter van Run, W. Paul Duprex, Judith M. A. van den Brand, Rik L. de Swart, Rory D. de Vries

**Affiliations:** 1 Division of Pathology, Faculty of Veterinary Medicine, Utrecht University, Utrecht, Netherlands; 2 Department of Viroscience, Erasmus MC, Rotterdam, Netherlands; 3 Viroclinics Xplore, Schaijk, Netherlands; 4 Center for Vaccine Research, University of Pittsburgh School of Medicine, Pittsburgh, Pennsylvania, USA; Stanford University School of Medicine, Stanford, California, USA

**Keywords:** distemper, morbillivirus, pathogenesis, raccoon, canine distemper virus

## Abstract

Raccoons are naturally susceptible to canine distemper virus (CDV) infection and can be a potential source of spill-over events. CDV is a highly contagious morbillivirus that infects multiple species of carnivores and omnivores, resulting in severe and often fatal disease. Here, we used a recombinant CDV (rCDV) based on a full-genome sequence detected in a naturally infected raccoon to perform pathogenesis studies in raccoons. Five raccoons were inoculated intratracheally with a recombinant virus engineered to express a fluorescent reporter protein, and extensive virological, serological, histological, and immunohistochemical assessments were performed at different time points post inoculation. rCDV-infected white blood cells were detected as early as 4 days post inoculation (dpi). Raccoon necropsies at 6 and 8 dpi revealed replication in the lymphoid tissues, preceding spread into peripheral tissues observed during necropsies at 21 dpi. Whereas lymphocytes, and to a lesser extent myeloid cells, were the main target cells of CDV at early time points, CDV additionally targeted epithelia at 21 dpi. At this later time point, CDV-infected cells were observed throughout the host. We observed lymphopenia and lymphocyte depletion from lymphoid tissues after CDV infection, in the absence of detectable CDV neutralizing antibodies and an impaired ability to clear CDV, indicating that the animals were severely immunosuppressed. The use of a wild-type-based recombinant virus in a natural host species infection study allowed systematic and sensitive assessment of antigen detection by immunohistochemistry, enabling further comparative pathology studies of CDV infection in different species.

**IMPORTANCE:**

Expansion of the human interface supports increased interactions between humans and peridomestic species like raccoons. Raccoons are highly susceptible to canine distemper virus (CDV) and are considered an important target species. Spill-over events are increasingly likely, potentially resulting in fatal CDV infections in domestic and free ranging carnivores. CDV also poses a threat for (non-human) primates, as massive outbreaks in macaque colonies were reported. CDV pathogenesis was studied by experimental inoculation of several species, but pathogenesis in raccoons was not properly studied. Recently, we generated a recombinant virus based on a full-genome sequence detected in a naturally infected raccoon. Here, we studied CDV pathogenesis in its natural host species and show that distemper completely overwhelms the immune system and spreads to virtually all tissues, including the central nervous system. Despite this, raccoons survived up to 21 d post inoculation with long-term shedding, supporting an important role of raccoons as host species for CDV.

## INTRODUCTION

Canine distemper virus (CDV), a member of the genus *Morbillivirus*, family Paramyxoviridae, is an enveloped negative-sense single-stranded RNA virus. Like its human analogue, measles virus (MV), CDV is highly contagious and can spread via the airborne route. CDV transmission also occurs via direct contact with an infected animal or with secretions through fighting, biting, or eating. Distemper was initially recognized as an infectious disease of dogs, but CDV infects a wide range of carnivorous and omnivorous host species and frequently progresses to severe and often fatal disease (1, 2). The virus has been described to easily jump the species barrier into marine mammals and peccaries (among other species) (3, 4). In the last decade, CDV also crossed into non-human primates, specifically causing large outbreaks in captive macaques (5 - 7). Raccoons (*Procyon lotor*), an omnivorous species that is highly prevalent in the USA and Canada, have also been identified as an important host species for CDV infection (8 - 10). CDV causes high mortality rates among raccoons, but widespread distribution in populations with surviving animals has also been reported, suggesting that raccoons are possible wildlife reservoirs for CDV (11, 12).

Ferrets (*Mustela putorius furo*) are highly susceptible to CDV and have been used as model system to study CDV pathogenesis (13 - 15). The pathogenesis of CDV was additionally studied in experimental settings in dogs, raccoon dogs (*Nyctereutes procyonoides*), foxes (*Vulpes vulpes*), and mink (*Neovison vison*). The mortality rates and development of histological lesions after infection were remarkably different between these species, with close to 100% mortality in ferrets and raccoon dogs, 40% in foxes, and no mortality observed in mink (2). The pathogenesis of wild-type CDV infection in raccoons has previously only been studied in a single experimental infection study. Two raccoons were inoculated with brain tissue from clinically ill raccoons captured in the wild and developed clinical symptoms similar to those observed in field cases, but no details on infection kinetics and histological lesions were reported (16).

The pathogenesis of distemper largely resembles that of measles in humans, with a number of critical differences. After CDV entry into the host by the respiratory or oral route, resident immune cells are infected via binding of the viral hemagglutinin (H) protein to the cellular receptor CD150 (also known as signaling lymphocyte activating molecule family member 1 or SLAMF1) (17). CDV is initially amplified in lymphoid tissues like bronchus-associated lymphoid tissues (BALTs) or lymph nodes directly draining the respiratory tract. After local amplification, CDV-infected immune cells spread through the circulation and lymphatic system, leading to a cell-associated viremia and dissemination of the infection to all peripheral lymphoid tissues. CDV targets several organ systems, including the respiratory system and the gastrointestinal (GI) tract. After widespread dissemination by immune cells, infection of epithelial cells is mediated by the cellular receptor nectin-4, which is part of the adherens junction and located at the basolateral cell membrane (18, 19). From this point onward, MV and CDV infection differ remarkably; MV infection is controlled by the adaptive immune system, whereas CDV infection often can no longer be controlled after overwhelming the immune system (20). This results in CDV-infected cells in almost all organs, including the central nervous system (CNS) (17) and various clinical signs, like biphasic fever, conjunctivitis, pneumonia, and encephalitis. In addition, infection of immune cells leads to severe lymphodepletion and subsequent immune suppression, increasing the risk of secondary infections (2, 15, 17). The end stages of disease (from recovery to fatal infections) are likely influenced by the capacity of the adaptive immune system to control CDV infection but can also be determined by host factors or co-infections.

Experimental infections to study the pathogenesis of distemper have mostly been performed with passaged virus isolates, like strains that were specifically adapted for infection of the CNS by passaging over brains (CDV^SH^), or viruses adapted to ferrets by serial passaging in that host (CDV^5804P^) (15, 21). Recently, a recombinant CDV directly based on a full-genome sequence detected in a naturally infected raccoon in Rhode Island (CDV^RI^), USA was generated for pathogenesis studies (10). This virus has minimal *in vitro* passage history and displays no cell-culture adaptations. To study the pathogenesis of this recombinant CDV, ferret infection studies were performed. As expected, CDV^RI^ infection started in myeloid and lymphoid cells, followed by infection of epithelial cells, resulting in systemic dissemination to multiple tissues and organs, especially those of the lymphatic system. High percentages of infected immune cells resulted in depletion of these cells both from circulation and from lymphoid tissues. The infection was lethal in 82% of the inoculated ferrets (22).

Here, we inoculated raccoons with a wild-type-based recombinant CDV to determine whether this is a promising recombinant strain to further study the pathogenesis of distemper. To perform an accurate assessment of the pathogenesis in the natural host, a virus engineered to additionally express a fluorescent reporter protein [rCDV^RI^Venus(6)] was used. Virological and serological assessments were performed on blood obtained from raccoons during the course of experimental infection; virological, histological, and immunohistochemical assessments were performed on tissues obtained at different time points post inoculation.

## RESULTS

### *Ex vivo* infection of raccoon white blood cells

To determine the susceptibility and permissiveness of raccoon WBC to morbillivirus infection, healthy raccoon WBC were stimulated with ConA to upregulate CD150 expression and subsequently inoculated with CDV [rCDV^RI^Venus(6])] at an MOI of 1. MV infection [rMV^KS^Venus(3)] and mock infection were included as controls. Infection was tracked and visualized by fluorescence microscopy, and the infection percentage was determined by flow cytometry at 24, 48, and 96 h post inoculation (hpi). Green fluorescent cells were detected as early as 24 hpi. Although ConA-stimulated raccoon WBC were susceptible to both rMV^KS^Venus(3) and rCDV^RI^Venus(6) infection, the cells were exclusively permissive to rCDV^RI^Venus(6) infection, reaching >50% infection at 96 hpi (Fig. 1; Fig. S2).

**Figure:**
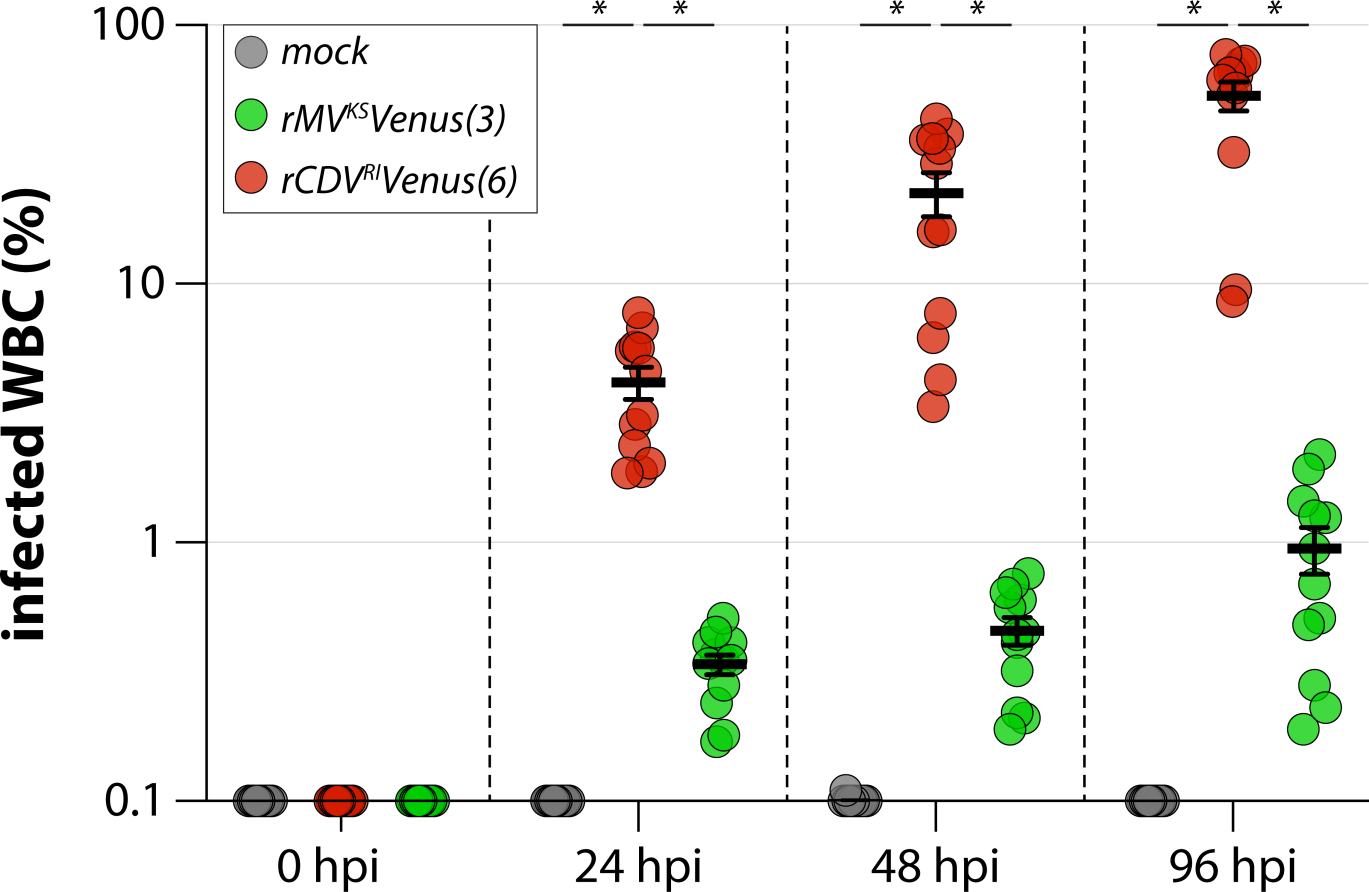
**FIG 1** *Ex vivo* infection of raccoon WBC. Infection percentage of raccoon WBC 24, 48, and 96 hpi with mock, rMV^KS^Venus(3), or rCDV^RI^Venus(6) measured by flow cytometry. The percentage of Venus-positive cells in the LIVE singlet gate was determined. Two identical experiments were combined in this graph, corresponding to the two experimental *in vivo* sessions. Duplicates were measured per donor animal (*N* = 6) at every time point, leading to 12 datapoints per virus per time point. Bar indicates the mean; whiskers indicate the standard error of the mean (SEM). *P*-values were estimated by one-way ANOVA performed per time point, asterisks denote *P* < 0.05. ANOVA, analysis of variance.

### Clinical signs

Five CDV-seronegative raccoons (*P. lotor*) were inoculated with 10^4^ TCID50 rCDV^RI^Venus(6) via IT inoculation to assess virus replication kinetics, tissue tropism, pathology, and virus-specific immune responses. Two clinical parameters were assessed after inoculation: body temperature (continuous monitoring via subcutaneously implanted probe) and lymphocyte counts (during every blood sampling). From 8 dpi onward, all animals developed a fever, which peaked around 17 dpi at +2°C. The fever did not resolve during the course of the experiment (Fig. 2A). Lymphocyte counts in peripheral blood decreased almost directly after inoculation, reaching lymphocyte levels of <20% relative to the starting levels from 10 dpi onward. Lymphocyte counts did not recover during the course of the experiment (Fig. 2B). We did not observe respiratory signs during the course of infection, but all animals displayed overt lethargy.

**Figure:**
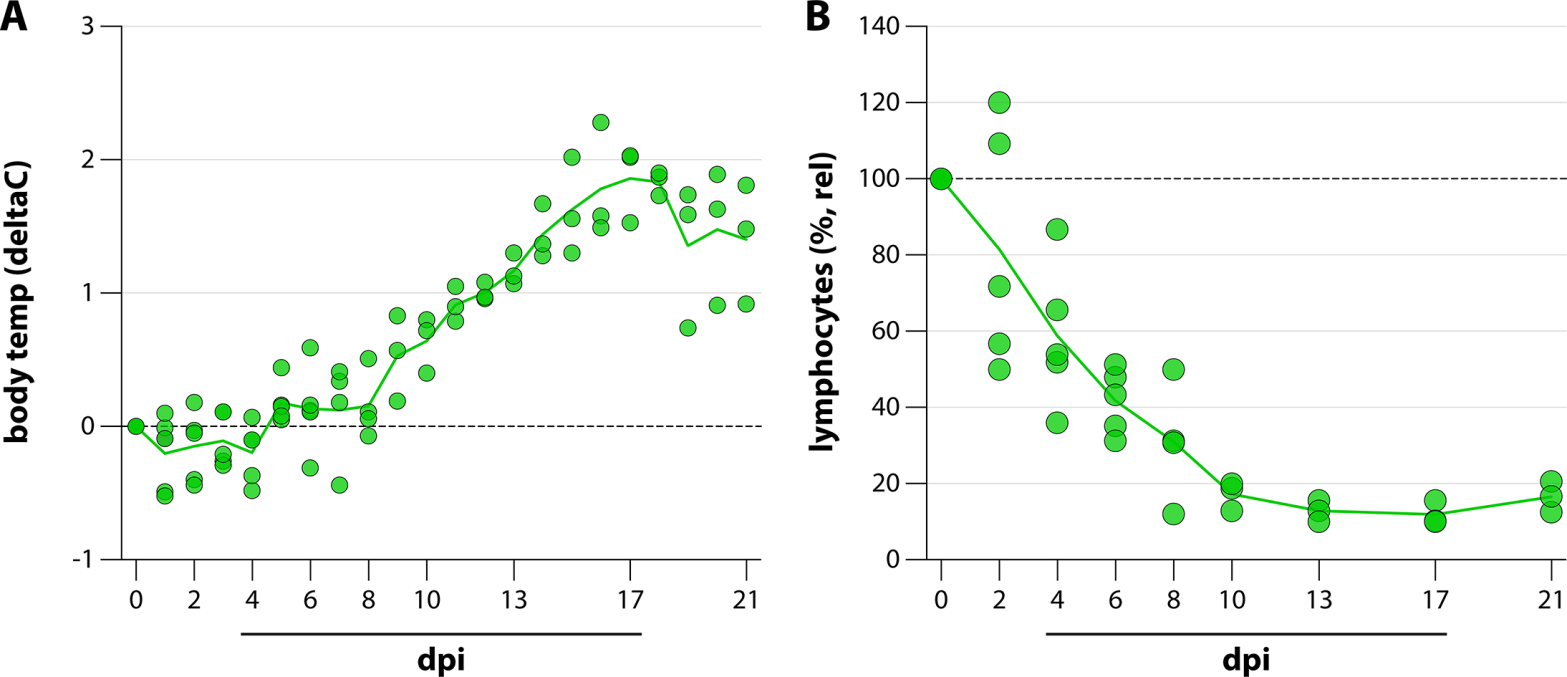
**FIG 2** Clinical parameters. During the course of the experiment, raccoons were assessed for two clinical parameters. (**A**) Body temperatures were measured by a subcutaneously implanted probe that obtained measurements every 10 min. Daily mean temperature difference relative to 0 dpi is shown. (**B**) Lymphocyte counts were measured every blood sampling. Relative counts compared with 0 dpi are shown. Symbols represent individual animals; lines connect the means per time point.

### Viremia and viral shedding

WBC collected during the course of the experiment were assessed for the presence of rCDV^RI^Venus(6)-infected cells by both virus isolation and flow cytometry. Virus isolation via titration of raccoon WBC on Vero cells stably expressing the CDV receptor dog SLAM (VDS cells) indicated the systemic presence of rCDV^RI^Venus(6)-infected cells from 4 dpi onward, which peaked at 8 dpi and declined toward 21 dpi. Infected cells could still be detected at the time point of necropsy, indicating that the immune system did not clear the infection (Fig. 3A). Infected cells were not detected in the uninfected control animal. Similar replication kinetics were observed when Venus-positive cells were quantified via flow cytometry (Fig. 3B; Fig. S3). rCDV^RI^Venus(6) shedding was detected in throat, nose, rectum, and eye swabs of all animals (Fig. 3C through F). Viral loads peaked earliest in the throat at 6 and 8 dpi, in a biphasic pattern where high levels of virus shedding were again observed at 21 dpi. This biphasic pattern was also observed in the rectum and eye swabs, the nose swabs displayed slightly different kinetics, with increasing viral titers up to 21 dpi.

**Figure:**
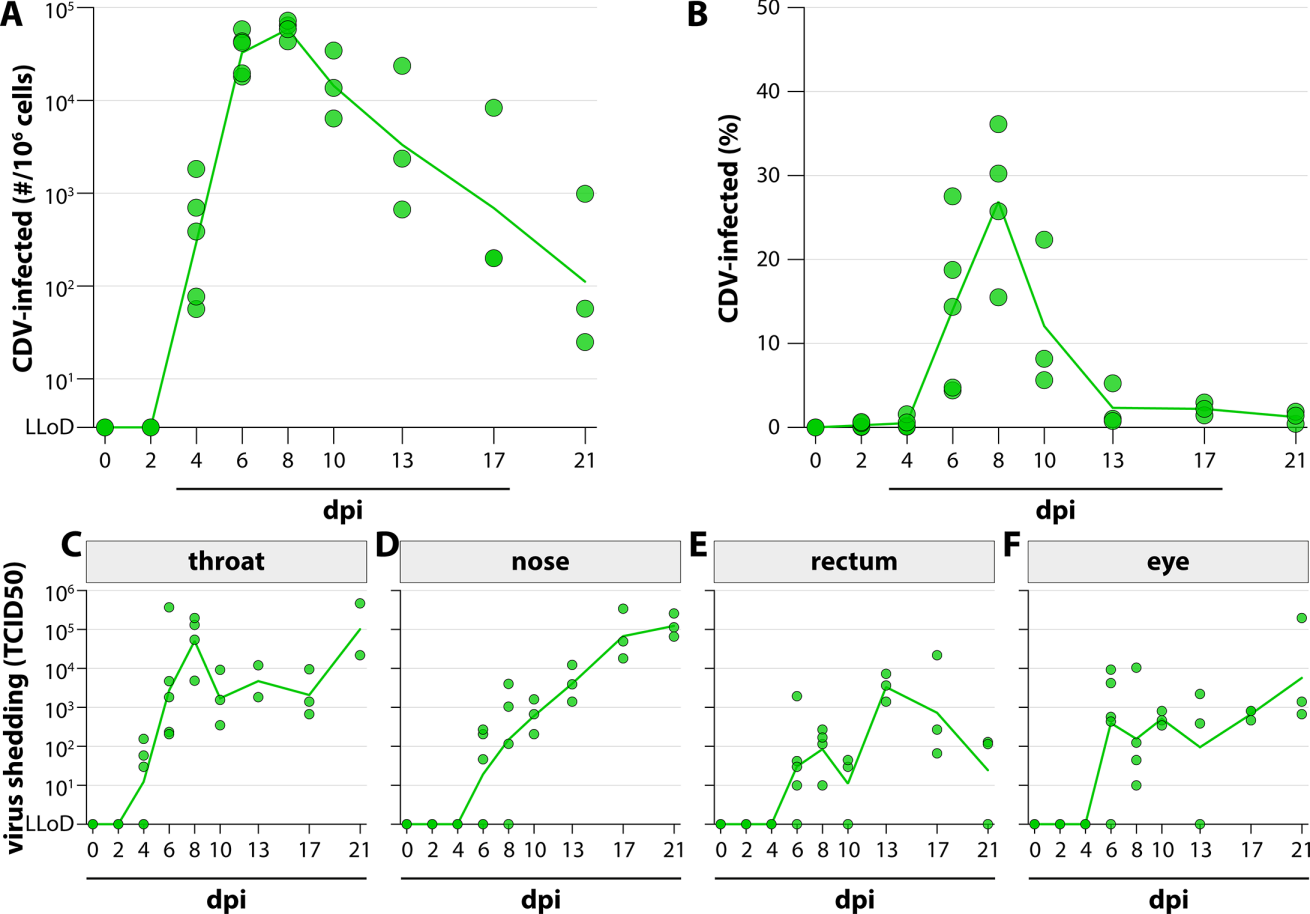
**FIG 3** rCDV^RI^Venus(6) systemic dissemination and shedding. Detection of rCDV^RI^Venus(6) in peripheral blood via (**A**) virus isolation or (**B**) flow cytometry. (**A**) WBC were titrated on VDS cells, viral loads were expressed as number of infected cells per 10^6^ total cells. (**B**) Infection percentage of raccoon WBC measured by flow cytometry. The percentage of Venus-positive cells in the LIVE singlet gate was determined. (**C–F**) Detection of rCDV^RI^Venus(6) shedding via endpoint titration of swab material on VDS. Viral loads were expressed as TCID50/mL. Viral shedding was measured in the (**C**) throat, (**D**) nose, (**E**) rectum, and (**F**) eyes. Symbols represent individual animals; lines connect the means (**B**) or geometric means (**A, C–F**) per time point.

### Viral tropism in peripheral blood

The phenotype of rCDV^RI^Venus(6)-infected cells was determined by flow cytometry on the basis of cell size (forward scatter [FSC]) and granularity (side scatter [SSC]). Lymphocytes, monocytes, granulocytes, and dendritic cell (DC)-like cells were identified on basis of similarity to human WBC FSC-SSC plots (Fig. 4A). In peripheral blood, lymphocytes were the predominant infected cell population (Fig. 4B). The percentage of infected lymphocytes peaked at 8 dpi (up to 80% of lymphocytes infected) and declined toward 21 dpi. Additionally, CDV-infected monocytes and DC-like cells, both from the myeloid lineage, were detected. However, infection percentages were lower and peaked around 20%. Inverse gating of the Venus-positive cells followed by phenotyping of the fractions within that population revealed a similar dominance for CDV infection of lymphocytes (Fig. 4C, representative sample at 8 dpi shown with 27% CDV-infected WBC). By determining the relative size of the different populations over time by flow cytometry, it was found that lymphocytes were depleted from peripheral blood (Fig. 4D, confirming the result in Fig. 1C).

**Figure:**
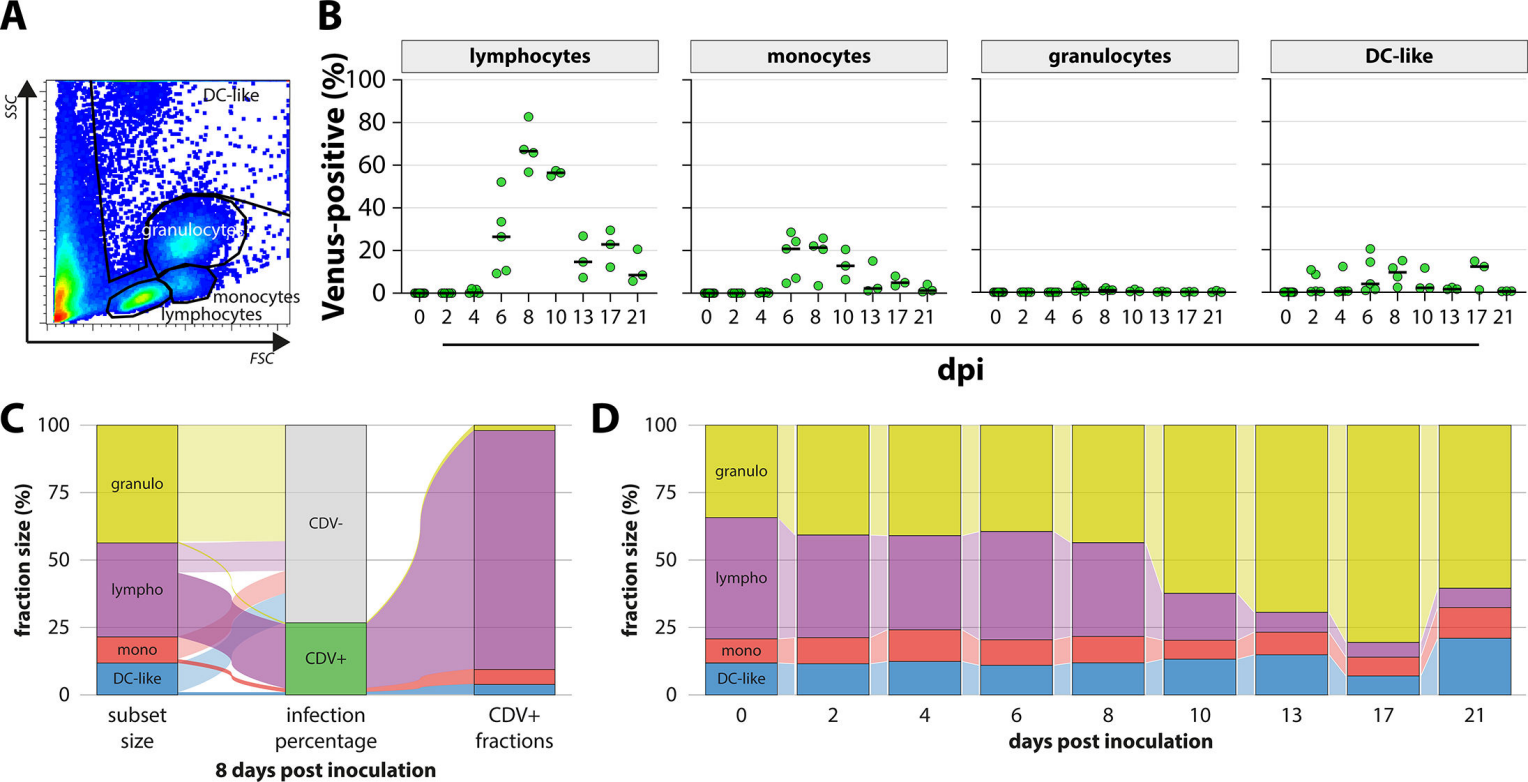
**FIG 4** Phenotype of rCDV^RI^Venus(6)-infected cells in peripheral blood. (**A**) Cells in peripheral blood were identified via flow cytometry on basis of FSC-SSC characteristics. Lymphocytes, monocytes, granulocytes, and DC-like cells are indicated. (**B**) Infection percentage of different cell types measured by flow cytometry. The percentage of Venus-positive cells in the indicated gate was determined. Symbols represent individual animals. (**C**) Phenotyping of CDV-infected cells on 8 dpi via inverse gating of Venus-positive cells within the LIVE singlet gate. The vast majority of the 27% Venus-positive cells in this representative raccoon was identified as lymphocytes. (**D**) Identification of populations via flow cytometry allowed for the determination of the relative population size at each time point. Mean population sizes of all available sample per time point are shown.

### Viral tropism at peak viremia

Necropsies were performed on the animals euthanized early after inoculation (6 and 8 dpi) to determine rCDV^RI^Venus(6) tropism at peak viremia. Based on direct excision and inflation of the lungs, followed by processing, counterstaining with a nuclear dye and confocal microscopy, rCDV^RI^Venus(6)-infected cells were observed in both the epithelium of the larger airways (Fig. 5A) as well as in the alveoli (Fig. 5B and C). Upon closer inspection of Venus-positive cells within the lungs, many of the clusters of infected cells were present in structures reminiscent of BALT (Fig. 5B arrows, Fig. 5C larger magnification). Outside the respiratory tract, macroscopically Venus-positive foci of cells were observed in all lymphoid tissues. As an example, Venus-positive foci are shown in the tracheobronchial lymph nodes (TBLN) (Fig. 5D arrows), the mesenteric LN (mesLN) (Fig. 5E arrows), and the tonsils (Fig. 5F arrows). Additionally, tertiary lymphoid structures were often found to contain Venus-positive foci, for example, the GALT (Fig. 5E asterisk). During necropsy, we generated single-cell suspensions from the tonsils, mandibular LN (manLN), retropharyngeal LN (RPLN), axillary LN (AXLN), thymus, TBLN, spleen, mesLN, and inguinal LN (ingLN). Additionally, we collected lymphocytes from the BAL, Peyer’s patches (PP), bone marrow (BM), and CSF. Venus-positive lymphoid cells were detected at all these locations by flow cytometry (Fig. 5G), although low infection percentages were observed in the BAL, BM, and CSF at 6 and 8 dpi.

**Figure:**
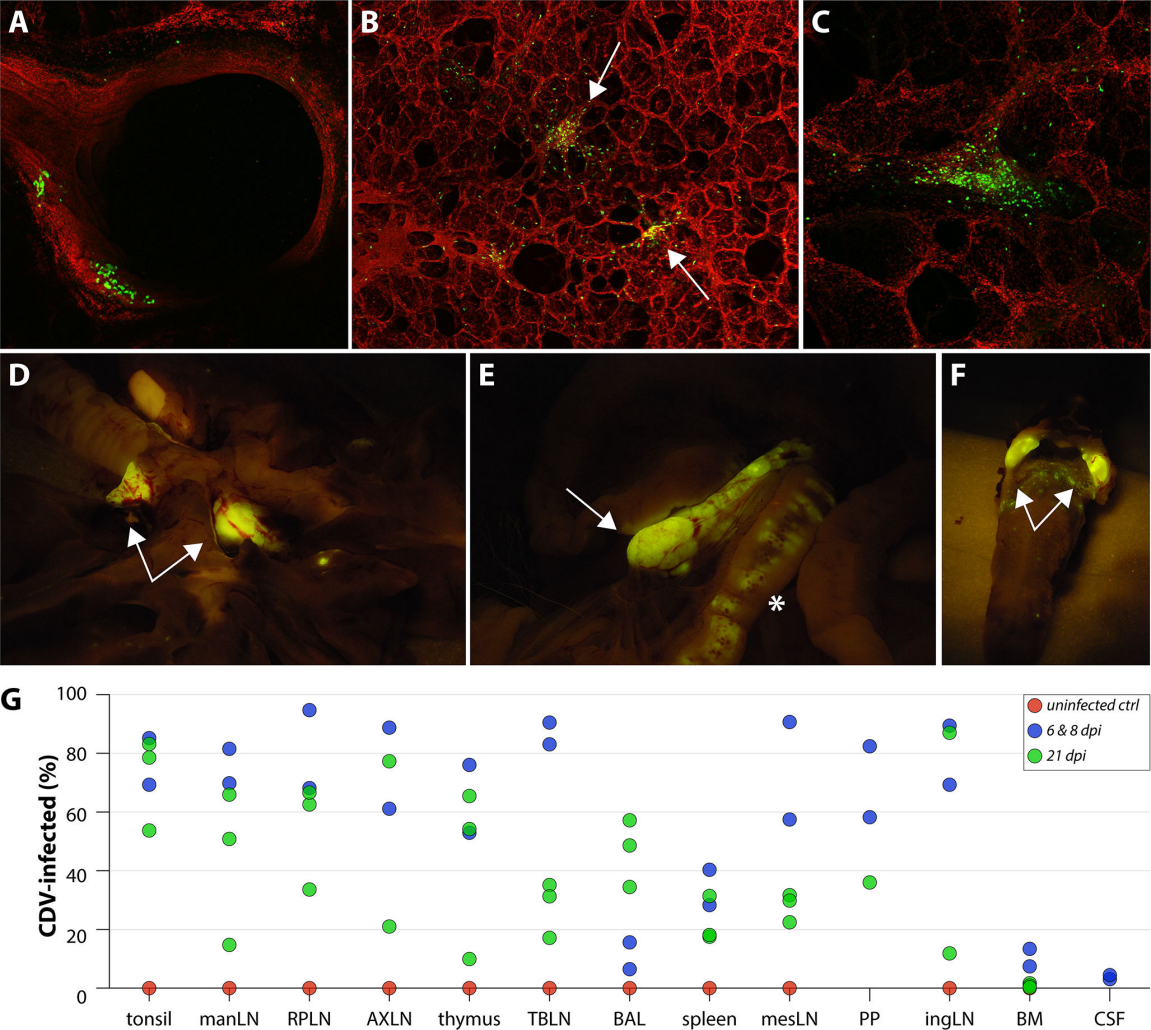
**FIG 5** Detection of Venus-positive cells at 6 dpi. (**A–C**) Samples from the respiratory tract (trachea, lungs) were screened for fluorescence after counterstaining of nuclei by confocal microscopy. At 6 dpi, Venus-positive cells were observed in the (**A**) larger airways and (**B, C**) lungs. Within the lungs, many foci of infected cells were observed in BALT structures indicated by arrows. (**D–F**) Macroscopic fluorescence in necropsies at 6 and 8 dpi was mainly observed in the lymphoid tissues, for example, TBLN (D, arrows), mesLN (E, arrow), GALT (E, asterisk), or tonsils (**F**). (**G**) rCDV^RI^Venus(6) infection percentages were measured in single-cell suspensions from different tissues via flow cytometry. Percentage Venus-positive cells within the LIVE singlet gate is shown. Symbols represent individual animals.

### Viral tropism at 21 dpi

Whereas at 6 and 8 dpi, rCDV^RI^Venus(6)-infected cells were mainly detected in the lymphoid tissues and structures, the infection had spread further into the periphery at 21 dpi. The lack of control by the immune system was demonstrated by the absence or low level of neutralizing antibodies detected at 21 dpi (Fig. S4). Upon necropsy, Venus-positive cells were still observed in many of the lymphoid organs (Fig. 6A and B, spleen and GALT/Peyer’s patches, respectively). Additionally, Venus-positive cells were readily observed in non-lymphoid organs, like the pancreas, kidney, urinary bladder, gall bladder, stomach, and bile duct (Fig. 6C through I), which did not contain fluorescent cells at 6 and 8 dpi. At this later time point, rCDV^RI^Venus(6) had also spread into the skin, observed by the presence of macroscopic fluorescence around the eyes and nose, on the skin of the abdomen, and on the footpads (Fig. 7A through C). As high percentages of infected cells were detected in the BAL at 21 dpi (Fig. 5G green symbols), it was not surprising that macroscopic fluorescence was readily detected in the nasal concha, the nasal septum, the trachea, and the lungs (Fig. 7D through F). Although difficult to assess macroscopically, fluorescence was also observed in the olfactory bulb (Fig. 7G) and brain (Fig. 7H). Flow cytometry of single-cell suspensions from lymphoid organs revealed fewer infected cells at 21 dpi, as compared with earlier time points (Fig. 5G), with the exception of the BAL. This fits well with the kinetics of infection, during which a combination of lymphopenia and lower numbers of CDV-infected cells were observed at later time points (Fig. 2B; Fig. 3A and B). Low numbers of CDV-infected cells were again observed in the CSF and BM at 21 dpi. No Venus-positive cells were detected in the uninfected control raccoon.

**Figure:**
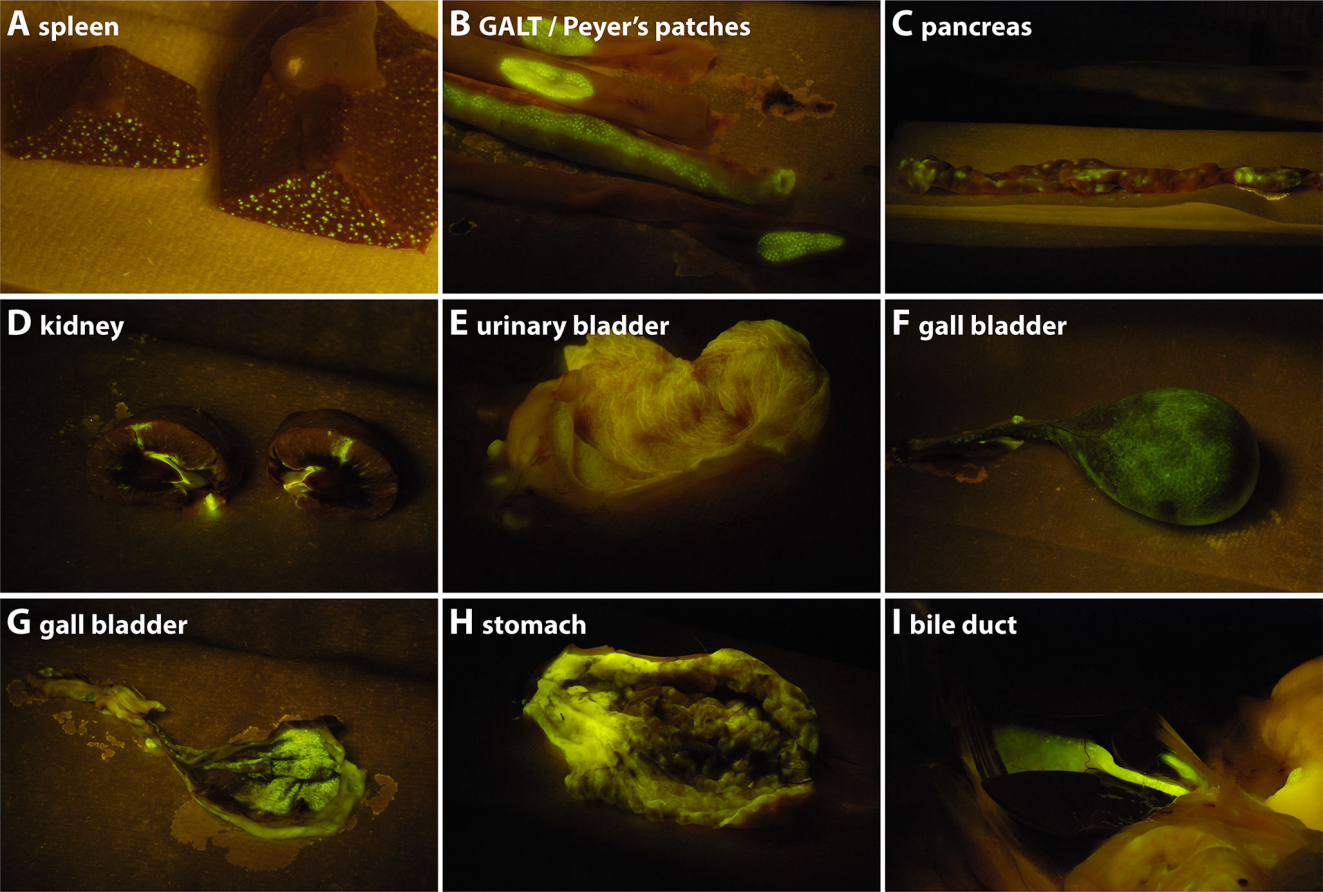
**FIG 6** rCDV^RI^Venus(6) spreads into the periphery at 21 dpi. (**A–I**) Macroscopic fluorescence in necropsies at 21 dpi was observed throughout the raccoon body. For example, we detected rCDV^RI^Venus(6)-infected cells in the (**A**) spleen, (**B**) GALT/Peyer’s patches, (**C**) pancreas, (**D**) kidney, (**E**) urinary bladder, (**F**) gall bladder outside, (**G**) gall bladder inside, (**H**) stomach, and (**I**) bile duct.

**Figure:**
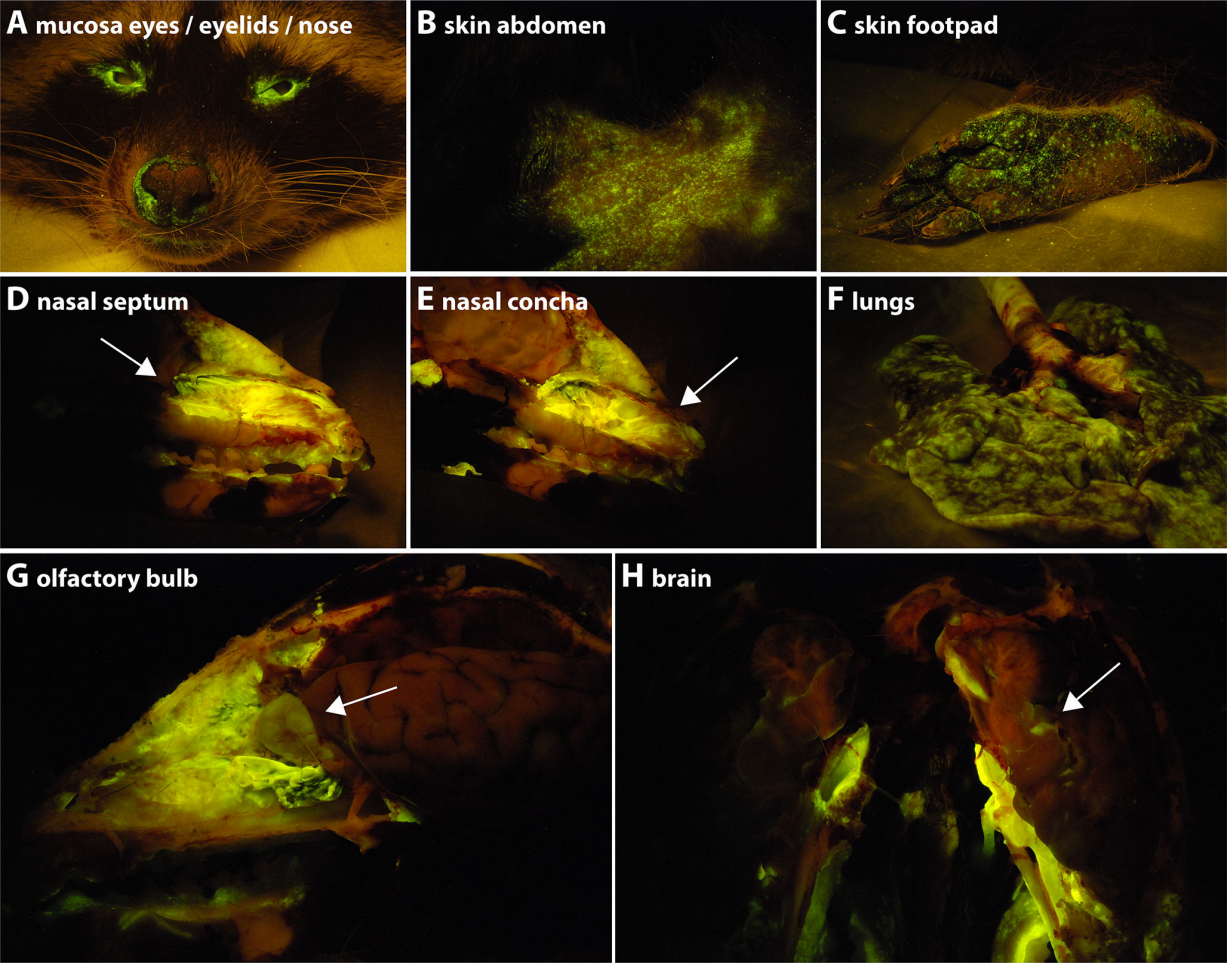
**FIG 7** rCDV^RI^Venus(6) in the skin, respiratory tract, and central nervous system at 21 dpi. (**A–C**) Macroscopic fluorescence in necropsies at 21 dpi was observed on the (**A**) mucosa of the eyes and eyelids, (**B**) skin of the abdomen, and (**C**) footpads. In the nose, macroscopic fluorescence was predominantly observed in the (**D**) septum (indicated by arrow) and (**E**) concha (indicated by arrow). (**F**) Macroscopic fluorescence was dispersed throughout the lungs. In the CNS, macroscopic fluorescence was observed in the (**G**) olfactory bulb (indicated by arrow) and (**H**) brain (indicated by arrow).

### Histopathology in lymphoid organs

Upon histopathological evaluation, lymphodepletion was observed in all lymphoid organs that were examined in detail (TBLN and ingLN, tonsil, spleen, BALT, and GALT) (Table S1). Lymphodepletion started at 6 dpi with an average score of 1 (*n* = 1 animal); at 8 dpi, mild-to-moderate lymphodepletion was observed in all lymphoid organs (*n* = 1 animal) (Fig. 8A, TBLN and BALT shown). Lymphodepletion was most severe at 21 dpi, especially apparent in the cortex of lymph nodes and in the spleen, for example, the ingLN (average scores of 2, 3) (Fig. 8A, ingLN and spleen shown). Necrosis, characterized by loss of tissue structure with karyorrhexis and karyolysis, was only observed (average score of 1) in the cortex of the TBLN and ingLN of one animal at dpi 21. Necrosis was not seen in any other organ, at any other time point. Inclusion bodies that are typically associated with morbillivirus infections were observed (23) and were about 2–4 µM in size, oval to round, hypereosinophilic, and located in the cytoplasm of lymphoid cells (mainly lymphocytes). Inclusion bodies were observed at 6 dpi in the TBLN and spleen, followed by the ingLN and tonsil at 8 dpi (spleen shown in Fig. S5A). Finally, a mild-to-moderate increase in numbers of macrophages was seen in the TBLN (cortex/paracortex) and ingLN (paracortex) at 8 dpi. Influx of eosinophils was also observed in the TBLN and ingLN (Table S1).

**Figure:**
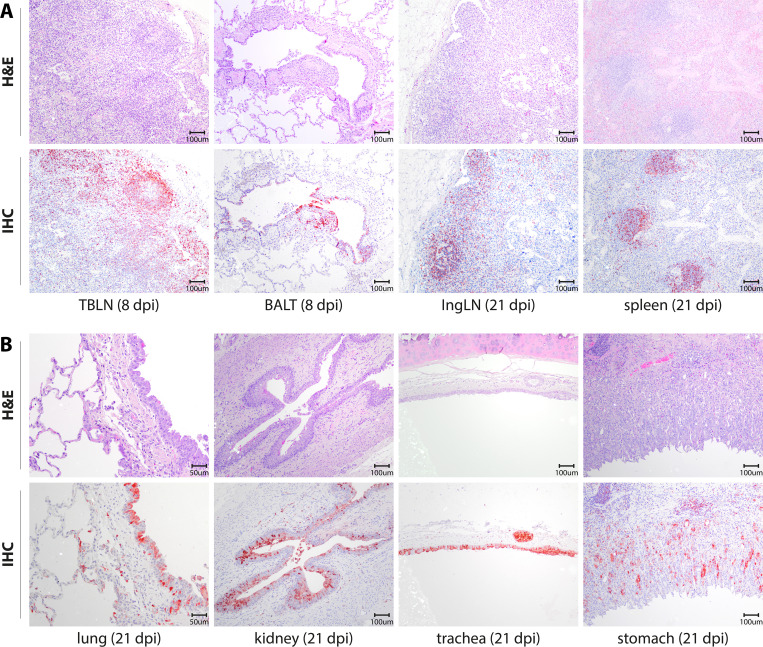
**FIG 8** Histological and immunohistochemical evaluation of lymphoid tissues and epithelia at 8 and 21 dpi. (**A**) Histopathology of TBLN (8 dpi), lungs (8 dpi), ingLN (21 dpi), and spleen (21 dpi) shown in H&E staining (top row) and virus antigen distribution (bottom row). (**B**) Histopathology of lung, kidney, trachea, and stomach (21 dpi) shown in H&E staining (top row) and virus antigen distribution (bottom row). Immunohistochemistry was performed with an anti-green fluorescent protein (GFP) antibody.

### Histopathology in epithelia

Inclusion bodies were observed in the cytoplasm of epithelial cells, at 6 dpi in the lung, followed by the esophagus at 8 dpi and the nasal septum, concha, trachea, primary bronchus, kidney, stomach, and ileum at 21 dpi (Table S2, kidney and lung shown in Fig. S5A). Inclusion bodies were predominantly observed in animals sacrificed at 21 dpi. Necrosis was not observed in any of the epithelial tissues examined. In several organs, small infiltrations of eosinophils were observed, including the lungs, nasal septum, gall bladder, kidney, stomach, and ileum. In the lung, moderate peribronchial inflammation was observed in one animal, with the inflammatory exudate existing predominantly of macrophages, neutrophils, and eosinophils at 21 dpi. In the kidney, mild-to-moderate lymphoplasmacytic interstitial nephritis with mild multifocal interstitial fibrosis and a few protein aggregates in the tubuli was observed in an animal euthanized at 21 dpi (Table S2). In another animal at 21 dpi, a focal cortical infarct was present in the kidney, characterized by a wedge-shaped area of interstitial lymphoplasmacytic nephritis, multifocal severe loss of tubular epithelium (necrosis), and a large amount of protein casts in the tubuli (Fig. S5D).

### Immunohistochemistry

Immunohistochemistry (IHC) was performed on slides sequential to the slides used for histopathology, and CDV was detected as diffuse to granular red antigen staining in the cytoplasm and occasionally in the nucleus of lymphocytes and epithelial cells. CDV was detected in small lymphocytes at 6 dpi in all lymphoid organs, and detection increased at 8 dpi in the LN and spleen with higher scores (between scores 1 and 2; scored on a scale from 0 to 3) (Table S1). CDV staining was still prominent at 21 dpi in all lymphoid organs examined with scores ranging from 1 to 3 in lymphocytes. Most virus antigen was detected in small lymphocytes in the cortex and paracortex of the ingLN of an animal euthanized at 21 dpi (score 3) (Fig. 8A). In the respiratory tract, CDV was detected at 6 dpi in the epithelium (average score of 1) of the nasal concha, the glandular epithelium of the trachea, the bronchial and glandular epithelium in the primary bronchus, and bronchiolar epithelium in the lung. At 8 dpi, the glandular epithelium (score of 1) and alveolar macrophages (score of 1) in the lung also stained positive for CDV. CDV was most prominently detected in the epithelial cells of animals sacrificed at 21 dpi, with epithelial scores ranging from 1 to 3, and overall detection of antigen was most extensive in the epithelial cells of the glands in the trachea (Fig. 8B; Table S2).

Outside the lymphoid tissues and respiratory tract, we evaluated several tissues for the presence of CDV-infected cells via immunohistochemistry. CDV was observed in epithelial cells in the skin, footpads, and eyelids (Fig. S5B). In the CNS, CDV-infected cells were detected in the brain, and the olfactory bulb in animals sacrificed on 21 dpi. In the brain, few lymphocytes in the meninges and choroid plexus stained positive (average score of 1). Several ependymal cells and several astrocytes and axons in the white and gray matter of the cerebrum stained positive in a single animal (score of 1) (Fig. S5C). In two animals, a few cells in de molecular layer of the cerebellum stained positive. In the olfactory bulb, a few positive cells (score of 1) were noted in all layers in two out of three animals euthanized at 21 dpi. Although CDV antigen was abundant in many other tissues, several were striking. In the gall bladder epithelium, positive virus antigen staining was observed from 6 to 21 dpi (with an average score of 1). In the GI tract, CDV-infected cells were detected in all animals at all time points in the epithelium and the submucosal glands of the esophagus. In the lining epithelium and glandular epithelium of the stomach, virus antigen was detected at 6 and 21 dpi (Fig. 8B). In the ileum, virus antigen in the epithelium was only detected at 21 dpi (average score of 1). Finally, in the kidney, CDV was detected in lymphocytes in the interstitium at 8 and 21 dpi (average score of 1) and in the epithelium of the pyelum at 21 dpi (score of 1–3) (Fig. 8B). In the cortical infarct of one animal with interstitial lymphoplasmacytic nephritis, virus antigen was detected in many lymphocytes (Fig. S5D).

## DISCUSSION

In this study, we report the tropism, virulence, and pathogenesis of a wild-type-based recombinant CDV strain in raccoons. CDV-infected white blood cells were detected in peripheral blood as early as 4 dpi. During necropsies at 6 and 8 dpi, CDV infection was predominantly present in the lymphoid tissues. At later time points, CDV infection was not cleared from lymphoid tissues but rather spread into the periphery. Simultaneously, viral loads remained high in the throat, nose, and eye swabs, indicating that CDV was not cleared efficiently. Whereas lymphocytes, and to a lesser extent myeloid cells, were the main target cells of CDV at early time points, CDV-infected epithelial cells were readily observed at 21 dpi. At this later time point, CDV-infected cells were observed in the lymphoid tissues, respiratory tract, skin, CNS, GI tract, urinary system (including the kidneys), gall bladder, and bile duct. Lymphopenia and lymphocyte depletion from lymphoid tissues after CDV infection in the absence of a detectable CDV neutralizing antibodies led us to conclude that CDV overwhelmed the raccoon immune system, leading to severe immune suppression and leaving animals susceptible for opportunistic infections.

Raccoons, highly susceptible to CDV infection, are an important target species for the virus and can be a potential source for spill-over events, making it a valuable model to study CDV pathogenesis and viral evolution in a natural, peridomestic host (1). Additionally, raccoons are known hosts for rabies and were recently shown to still be the primary host species for this disease in the USA (24), despite massive rabies bait vaccinations campaigns. Interestingly, as both rabies and CDV can present with neurological signs (including ataxia and disorientation) (25), it could be difficult to distinguish these diseases at first sight. A recent report additionally indicated that raccoons thought to be drunk from eating fermented crab apples were actually sick with distemper. The ongoing expansion of the human interface supports increased interactions between humans and peridomestic species like raccoons, but also skunks, coyotes, and foxes. Therefore, spill-over events with viruses like CDV and rabies virus are increasingly likely and could more often result in fatal infections in domestic and free ranging carnivores but also pose a threat for spill-over events into (non-human) primates.

Previous studies often used recombinant CDVs that were tissue- or host adapted, potentially resulting in different clinical presentation and disease progression *in vivo* (14, 26). Here, we used recombinant CDV^RI^ based on a sequence directly obtained from a naturally infected raccoon. It was recently shown in experimental infections of ferrets that this virus retains its lymphotropic and epitheliotropic nature but was less neurovirulent than the recombinant strains described so far in literature (22). Here, we show that the pathogenesis of distemper caused by rCDV^RI^ in raccoons resembles the pathogenesis observed in ferrets. However, in the majority of ferrets, experimental infection with rCDV^RI^Venus(6) was fatal within 20 dpi, whereas in our study, no raccoons reached their humane endpoints before 21 dpi. In the case of measles, which is usually self-limiting, replication peaks around 9 dpi (27, 28), whereas in lethal infections in ferrets, replication peaked at 6 dpi (22). We speculate that differences in disease outcome, but also the overwhelming of the immune system, between measles and distemper are likely due to differences in virus replication kinetics (20). Morbilliviruses of other target species follow similar replication kinetics, for example, peste des petits ruminant virus in goats and the non-fatal feline morbillivirus (FeMV) in cats. The low levels of infected WBC in peripheral blood after FeMV infection (<0.5%) are reflected in the low case fatality rates after acute infection, which further supports the role of replication kinetics on disease outcome (29, 30). Based on the rapid virus replication, persistent viremia, invasion of the CNS, increasing viral shedding (and no clearance), absent or low serological responses, and unresolved lymphopenia we observed in raccoons, we speculate that none of the animals would have survived over the course of a longer study period; however, longer-term studies are required to confirm this. Infection of the CNS in raccoons observed in this study was similar to that observed in ferrets, as in both species CDV was observed in the olfactory bulb, meninges, and choroid plexus.

The experimentally infected raccoons in our study presented with lesions similar to those observed in naturally infected raccoons (12, 16, 31). In our study, massive CDV infection of the lungs was observed, which increased between 6 and 21 dpi based on Venus-positive cells in the lungs and BAL; however, only mild pathological lesions were observed. One of the raccoons developed a mild-to-moderate bronchointerstitial pneumonia at 21 dpi, while studies of natural infections describe an encephalitis and necrotizing bronchointerstitial pneumonia (12, 16, 31), with occasional pulmonary hemorrhage and edema (16). Additionally, the presence and location of eosinophilic cytoplasmic inclusion bodies in different organs were similar between the raccoons in our study and naturally infected raccoons, and lymphodepletion was shown here and previously described in natural infections (12, 16, 31). Strain-to-strain differences could explain differences between our experimentally infected raccoons, but we cannot exclude that this is due to the use of a recombinant virus expressing Venus.

In dogs, several organ systems are affected by CDV, including the respiratory tract, CNS, GI tract, urinary system, lymphoid organs, endocrine organs, and the cutaneous system (17). As for the respiratory tract, an interstitial serofibrinous and hemorrhagic pneumonia has been previously described in dogs, raccoon dogs, and foxes, and a suppurative to necrotizing bronchopneumonia was described in African wild dogs. Compared with these species infected with CDV, lung lesions in the raccoons in our study were not as obvious; mild broncho-interstitial pneumonia was observed in only one raccoon, no clear lesions were observed in the other animals. The infection resembled what was previously observed in mink (2). As for the GI tract, gastroenteritis was observed in dogs, which we also observed in the experimentally infected raccoons. However, the changes were mild and concerned an eosinophilic gastroenteritis. We did not observe polioencephalitis and demyelinating leukoencephalomyelitis in raccoons, which have been described in dogs and foxes (1). Lymphodepletion in the spleen and lymph nodes was previously described in CDV-infected raccoon dogs, foxes, and mink, with mink showing least severe depletion. Although an exact quantitative comparison is impossible to make, all raccoons in our study presented with lymphopenia in peripheral blood and lymphodepletion from lymphoid organs.

In summary, our study shows how inoculation of raccoons with a wild-type-based recombinant CDV led to widespread infection of lymphoid, myeloid, and epithelial cells in a large range of organs, including invasion of the CNS. The severe lymphodepletion and overwhelming of the immune system prevented the production of CDV neutralizing antibodies. The major novelty of this study is determined by the use of a raccoon isolate-based recombinant virus expressing a fluorescent reporter protein [rCDV^RI^Venus(6)] in a natural host species infection study, which allowed systematic and sensitive assessment of clinical signs in association with macroscopic imaging, flow cytometry, and antigen detection by IHC. As infection in raccoons with this recombinant wild-type CDV resembles a natural infection with CDV, we conclude that this virus is a promising recombinant strain to further study the pathogenesis of distemper.

## MATERIALS AND METHODS

### Cells and viruses

Vero cells stably expressing the CDV receptor dog SLAM (VDS) (kind gift of Dr. Y. Yanagi, Kyushu University, Fukuoka, Japan) were cultured as described previously (32). To obtain primary raccoon white blood cells (WBC), blood samples were collected from racoons in Vacuette tubes (Greiner) containing K_3_EDTA as an anticoagulant. Red blood cells were lysed with red blood cell lysis buffer (Roche, Basel, Switzerland), washed, and WBC were resuspended in complete RPMI-1640 medium (Gibco Invitrogen, Carlsbad, CA, USA) supplemented with 2 mM L-glutamine, 10% (vol/vol) heat-inactivated fetal bovine serum (FBS), penicillin (100 U/mL), and streptomycin (100 µg/mL). The MV strain KS is a wild-type genotype B3 virus isolated from peripheral blood mononuclear cells collected in 1997 from a severe measles case in Khartoum Sudan (GenBank accession number HM439386) (33). The recombinant virus expressing Venus from the third position in the MV genome was generated as described previously (34). Virus stocks were grown in B lymphoblastic cell lines (B-LCL). The CDV strain Rhode Island (RI) virus was generated from an entirely synthetic molecular clone designed using the genomic sequence from a clinical isolate obtained from a free-ranging raccoon with distemper (GenBank accession number KU666057), as described previously (10). Virus stocks were grown in VDS cells. All stocks tested negative for contamination with *Mycoplasma* species. Virus titers were determined by endpoint titration in Vero cells expressing human SLAM or VDS cells and expressed in TCID50/mL. Susceptibility of raccoon WBC to rCDV^RI^Venus(6) infection was determined *ex vivo*. To this end, WBC were isolated from the raccoons used for the *in vivo* experiment prior to start. Concanavalin (ConA)-stimulated raccoon WBC were inoculated with rCDV^RI^Venus(6) or rMV^KS^Venus(3) at a multiplicity of infection (MOI) of 1 for 1 h, washed, and subsequently cultured for 96 h. Susceptibility of raccoon WBC was analyzed directly by detection of fluorescent reporter proteins by confocal laser scanning microscopy (CLSM) with an LSM700 system fitted on an Axio Observer Z1 inverted microscope (Zeiss) and by flow cytometry on a FACS Lyric (BD Biosciences).

### Animal study design

Six CDV-seronegative raccoons (*P. lotor*) were used for the infection studies (five infected, one control). A temperature probe was implanted subcutaneously before the beginning of the experiments to monitor body temperature noninvasively. The raccoon inoculation experiment was performed in two sessions. In the first session, *N* = 3 raccoons were inoculated with 10^4^ TCID50 rCDV^RI^Venus(6) via intra-tracheal (IT) inoculation by direct instillation of virus suspension (5 mL in phosphate-buffered saline [PBS]) into the lower respiratory tract after intubation with a flexible catheter and euthanized at a late time point (21 d post inoculation [dpi]) to assess virus replication kinetics, tropism in the periphery, pathology and virus-specific immune responses. In the second session, *N* = 2 raccoons were inoculated with 10^4^ TCID50 rCDV^RI^Venus(6) via IT inoculation and euthanized at the peak of virus replication (6 and 8 dpi) to study tissue tropism and pathology. One uninfected control raccoon was included in this second session of the study. Back-titration of the suspension used for inoculation confirmed the infectious dose. Following inoculation, raccoons were sampled regularly and were euthanized at 6, 8, or 21 dpi (Fig. S1). All analyses were performed with data from all *N* = 5 CDV-inoculated animals combined.

### Clinical specimen and detection of CDV-infected cells

Blood samples were collected in Vacuette tubes (Greiner Bio-One, Kremsmünster, Austria) containing K_3_EDTA as an anticoagulant. Lymphocyte counts were obtained using an automated counter (pocH-100iV; Sysmex). WBC were obtained by lysis of whole blood with red blood cell lysis buffer (Roche, Basel, Switzerland) as described above. Cells were counted using a hemacytometer and used directly for flow cytometry and virus isolation. The percentages of infected cells were determined by the detection of the fluorescent reporter protein Venus by flow cytometry. Isolation of rCDV^RI^Venus(6) was performed on VDS cells using an infectious center test as previously described (35). Virus isolations were monitored for cytopathic effect (CPE) by microscopy after co-cultivation with VDS for 3–6 d, and results were expressed as the number of virus-infected cells/10^6^ total cells. Throat and rectal swabs (cytobrush plus; Medscand Medical), and nose and eye swabs (polyester-tipped minitip urethral swab; Copan) were collected in transport medium (Eagle’s minimal essential medium with Hanks’ salts, supplemented with lactalbumin enzymatic hydrolysate, penicillin, streptomycin, polymyxin B sulfate, nystatin, gentamicin, and glycerol) and frozen at −80°C. After being thawed, samples were vortexed, the swab was removed, and the remaining transport medium was used for virus isolation. Isolation of rCDV^RI^Venus(6) was performed on VDS cells using an infectious center test as previously described (35). Virus isolations were monitored for CPE by microscopy after co-cultivation with VDS cells for 3–7 d, and results were expressed as TCID50/mL.

### Necropsy

Raccoons were euthanized by exsanguination under deep ketamine/medetomidine anesthesia. Macroscopic detection of Venus was performed with an LED lamp and the appropriate filters and photographed with an SLR camera as described previously (27). Post euthanasia, cerebrospinal fluid (CSF) was obtained by lumbar puncture. A bronchoalveolar lavage (BAL) was performed post-mortem by direct infusion of phosphate-buffered saline (10 mL) into the right-hand side of the lung. BAL cells were resuspended in culture medium with supplements as described above, counted, and used directly for flow cytometry. The infection percentages of BAL cells were determined by the detection of Venus by flow cytometry. During necropsy, multiple internal organs and tissues from the central nervous system, respiratory tract, digestive tract, and lymphatic system were harvested and screened directly for expression of fluorescent reporter proteins by CLSM. The left lung was inflated with 2% (wt/vol) low melting point agarose before being sliced and screened, as described previously (13, 34, 36). Lung slices selected for direct CLSM imaging were counterstained with TO-PRO3. After screening, non-lymphoid tissues were transferred to 10% neutral-buffered formalin (FA). Lymphoid tissues were initially collected in PBS. Half lymph nodes were also transferred to 10% neutral-buffered FA, the other half was used for preparation of single-cell suspensions using cell strainers with a 100-µm pore size (BD Biosciences, Erembodegem, Belgium) and directly used for flow cytometry. The infection percentages of single-cell suspensions were determined by flow cytometry.

### Virus neutralization assay

Virus-neutralizing (VN) antibody responses were measured by an endpoint neutralization assay. Briefly, serial 2-log dilutions (starting at 2^−2^) of heat-inactivated serum samples were incubated in triplicate with rCDV^RI^Venus(6) for 1 h at 37°C in 96-well flat-bottom plates. Subsequently, trypsinized VDS cells were added at a concentration of 1 × 10^4^ cells/well. Plates were incubated for 5–7 d at 37°C and visually monitored for fluorescence and/or CPE. VN titers were calculated as the 50% endpoint of triplicate measurements using the method of Reed and Muench (37).

### Histology and immunohistochemistry

After fixation in 10% formalin, 3-µm paraffin sections were deparaffinized, and antigens were retrieved by boiling slides for 15 min in citric acid buffer (10 mM, pH 6.0). Sections were incubated with the anti-GFP antibody for 1 h at RT. Binding of the primary antibody was detected using a biotinylated goat anti-rabbit Ig (DAKO), after which tissue sections were incubated with ABComplex-HRP (DAKO) for 30 min. Peroxidase was revealed using 3-Amino-9-ethyl-carbazole (AEC, Sigma), resulting in a bright red precipitate. In each staining procedure, an isotype control was included as a negative control, and for each section, a consecutive hematoxylin and eosin (H&E) was generated for histological evaluation.

To evaluate histopathological changes over time, a semiquantitative assessment was performed on a selection of tissues obtained during necropsy: tracheobronchial and inguinal lymph node, tonsil, spleen, nasal septum, concha, trachea, primary bronchus, lung, brain, olfactory bulb, gall bladder, esophagus, stomach, ileum, and kidney. The lesions were scored according to the following method: for lymphodepletion we scored: 0, none; 1, mild (depletion of 1%–10% of the cells); 2, moderate (depletion of 11%–40% of the cells); 3, severe (depletion of >40% of the cells). The extent of necrosis was scored: 0, none; 1, mild; 2, moderate; 3, severe. The severity of inflammation was scored: 0, no inflammatory cells; 1, few inflammatory cells; 2, moderate numbers of inflammatory cells; 3, large numbers of inflammatory cells. The inflammatory cell type which was most present was scored: N, neutrophil; M, macrophage; L, lymphocyte; P, plasma cell; Eo, Eosinophil. Additionally, the anatomical location of inflammation was noted in order to assess whether the presence of inflammation was associated with lesions. Cytoplasmic and/or intranuclear inclusion bodies were scored: 0, none; 1, present in 1%–10% of cells; 2, 11%–40% of cells; 3, >40% of cells. If applicable, for epithelial necrosis, we scored: 0, none; 1, 1%–10% of cells; 2, 11%–40% of cells; 3, >40% of cells. Slides were examined without knowledge of the identity of the animals. For every slide, 10 randomly chosen 10× objective fields were scored. For each scored parameter, the mean score (rounded up) of the 10 fields provided the average percentage score for that parameter in that organ.

To evaluate CDV tropism in several organs over time, a semiquantitative assessment of CDV-infected cells detected by immunohistochemistry was performed in the tracheobronchial and inguinal lymph node, tonsil, spleen, nasal septum, concha, trachea, primary bronchus, lung, brain, olfactory bulb, gall bladder, esophagus, stomach, ileum, and kidney. For every organ, 10 randomly chosen fields of view (using a 10× objective) were examined by light microscopy, with every positive cell type in each organ being scored separately. The percentage of positively staining cells was estimated per field as follows: 0, 0%; 1, 1%–10%; 2, 11%–40%; 3, >40%. The average of 10 fields was calculated to provide a total percentage score (rounded up) per cell type per organ. Slides were examined without knowledge of the identity of the animals.

### Statistical analysis

Statistical analysis was performed with GraphPad Prism 9.5.0. Results of statistical analyses are indicated in the figure legends.

## SUPPLEMENTAL MATERIAL

Supplementary Material msphere.00144-23-s0001.pdf Tables S1-S2, Figures S1-S5.

ASM does not own the copyrights to Supplemental Material that may be linked to, or accessed through, an article. The authors have granted ASM a non-exclusive, world-wide license to publish the Supplemental Material files. Please contact the corresponding author directly for reuse.
