# Supplementary material for: Inoculation of raccoons with a wild-type-based recombinant canine distemper virus results in viremia, lymphopenia, fever, and widespread histological lesions
Source: mSphere. 2023 Jun 14;8(4):e00144-23. doi: 10.1128/msphere.00144-23 (PMC10449507; doi:10.1128/msphere.00144-23)
Supplement: Supplementary Material — Tables S1-S2, Figures S1-S5. [file msphere.00144-23-s0001.pdf]

Supplemental Material to

Inoculation of raccoons with a wildtype-based recombinant canine distemper virus results in viremia, lymphopenia, fever and widespread histological lesions

Dagmar Roelofs[a]*, Katharina S. Schmitz[b]*, Geert van Amerongen[c], Laurine C. Rijsbergen[b], Brigitta M. Laksono[b], Anouskha D. Comvalius[b], Sham Nambulli[d], Linda J. Rennick[d], Peter van Run[b], W. Paul Duprex[d], Judith M.A. van den Brand[a], Rik L. de Swart[b$#], and Rory D. de Vries[b]

[a] Division of Pathology, Faculty of Veterinary Medicine, Utrecht University, Utrecht, the Netherlands

[b] Department of Viroscience, Erasmus MC, Rotterdam, the Netherlands

[c] Viroclinics Xplore, Schaijk, Netherlands

[d] Centre for Vaccine Research, University of Pittsburgh School of Medicine, Pittsburgh, USA

* equal contributions: Dagmar Roelofs and Katharina Schmitz contributed equally to this work. Author order was determined alphabetically.

[#] Correspondence to: Dr. Rik L. de Swart, Department of Viroscience, Erasmus MC, Dr. Molewaterplein 40, 3015 GD Rotterdam, the Netherlands, T: +31107044280; E: r.deswart@erasmusmc.nl

[$] Current address: Department of Virology, Wageningen Bioveterinary Research, Lelystad, the Netherlands

Table S1. Overview of immunohistochemistry and histology in lymphoid tissues. Median scores and range of IHC, lymphodepletion, inclusion bodies and necrosis in lymphoid organs. IHC scores: 0 , none; 1, 1-10% of cells positive; 2, 11-40% of cells positive; 3, >40% of cells positive. Lymphodepletion scores: 0 , none; 1, mild; 2, moderate; 3, severe. Inclusion body scores: 0 , none; 1, 1-10% of cells; 2, 11-40% of cells; 3, >40% of cells. Necrosis scores: 0 , none; 1, mild; 2, moderate; 3, severe.

			**DPI**	
**Organs**	**Subject**	**6 (N=1)**	**8 (N=1)**	**21 (N=3)**
TBLN	IHC	1 (1)	2 (2)	1,5 (1,5)
	Lymphodepletion	0 (0)	2 (2)	1,75 (1-2,5)
	Inclusion bodies	1 (1)	1 (1)	1 (1)
	Necrosis	0 (0)	0 (0)	0,5 (0-1)
IngLN	IHC	1 (1)	2 (2)	1,5 (1-3)
	Lymphodepletion	0,5 (0-1)	1 (1)	2,5 (1-2,5)
	Inclusion bodies	0 (0)	0 (0)	1 (1)
	Necrosis	0 (0)	0 (0)	0 (0-1)
Tonsil	IHC	1 (1)	1 (1)	1,5 (1-2)
	Lymphodepletion	0 (0)	1 (1)	1,5 (1-2)
	Inclusion bodies	0 (0)	1 (1)	1 (1)
	Necrosis	0 (0)	0 (0)	0 (0)
Spleen	IHC	1 (1)	1,5 (1)	1,5 (1-2)
	Lymphodepletion	1 (1)	1 (1)	1 (0-1)
	Inclusion bodies	1 (1)	1 (1)	1 (1-2)
	Necrosis	0 (0)	0 (0)	0 (0)

Table S2. Overview of immunohistochemistry and histology in the respiratory tract. Median scores and range IHC, inclusion bodies and necrosis in the respiratory tract. AT1: type 1 pneumocyte, AT2: type 2 pneumocyte, AM alveolar macrophage.

IHC scores: 0 , none; 1, 1-10% of cells positive; 2, 11-40% of cells positive; 3, >40% of cells positive. Inclusion body scores: 0 , none; 1, 1-10% of cells; 2, 11-40% of cells; 3, >40% of cells. Necrosis scores: 0 , none; 1, mild; 2, moderate; 3, severe.

		DPI		
**Organs**	**Subject**	**6 (N=1)**	**8 (N=1)**	**21 (N=3)**
Nasal septum	IHC nasal epithelium	0 (0)	0 (0)	1 (1)
	IHC glandular epithelium	0 (0)	0 (0)	1 (0-1)
	Inclusion bodies	0 (0)	0 (0)	1 (0-1)
	Necrosis	0 (0)	0 (0)	0 (0)
Nasal concha	IHC conchal epithelium	1 (1)	0 (0)	1 (1-2)
	IHC glandular epithelium	0 (0)	0 (0)	0 (0)
	Inclusion bodies	0 (0)	0 (0)	1 (1-2)
	Necrosis	0 (0)	0 (0)	0 (0)
Trachea	IHC tracheal epithelium	0 (0)	0 (0)	1 (1-3)
	IHC glandular epithelium	1 (1)	1 (1)	2 (2-3)
	Inclusion bodies	0 (0)	0 (0)	1 (0-2)
	Necrosis	0 (0)	0 (0)	0 (0)
Primary bronchus	IHC bronchial epithelium	1 (1)	1 (1)	1 (1-2)
	IHC glandular epithelium	1 (1)	0 (0)	1 (1-3)
	Inclusion bodies	0 (0)	0 (0)	1 (1-20
	Necrosis	0 (0)	0 (0)	0 (0)
Lung	IHC bronchial/bronchiolar epithelium	1 (1)	1 (1)	1 (1-2)
	IHC glandular epithelium	0 (0)	1 (1)	2 (0-2)
	IHC AT1	0 (0)	0 (0)	1 (1)
	IHC AT2	0 (0)	0 (0)	1 (0-1)
	IHC AM	0 (0)	1 (1)	1 (1-2)
	Inclusion bodies	1 (1)	0 (0)	1 (1)
	Necrosis	0 (0)	0 (0)	0 (0-1)

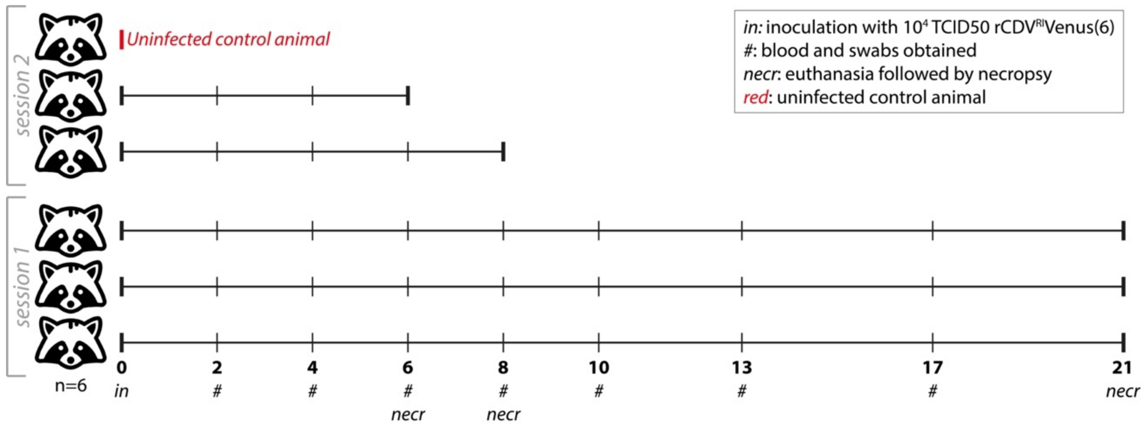

Figure S1. Animal study design. Animal experiment was performed in two sessions with N=3 raccoons. In the first session raccoons were euthanized at 21 dpi, the second session focused on early time points (6 and 8 dpi). The second session contained N=1 uninfected control animal (red). Blood and swabs were obtained at regular intervals.

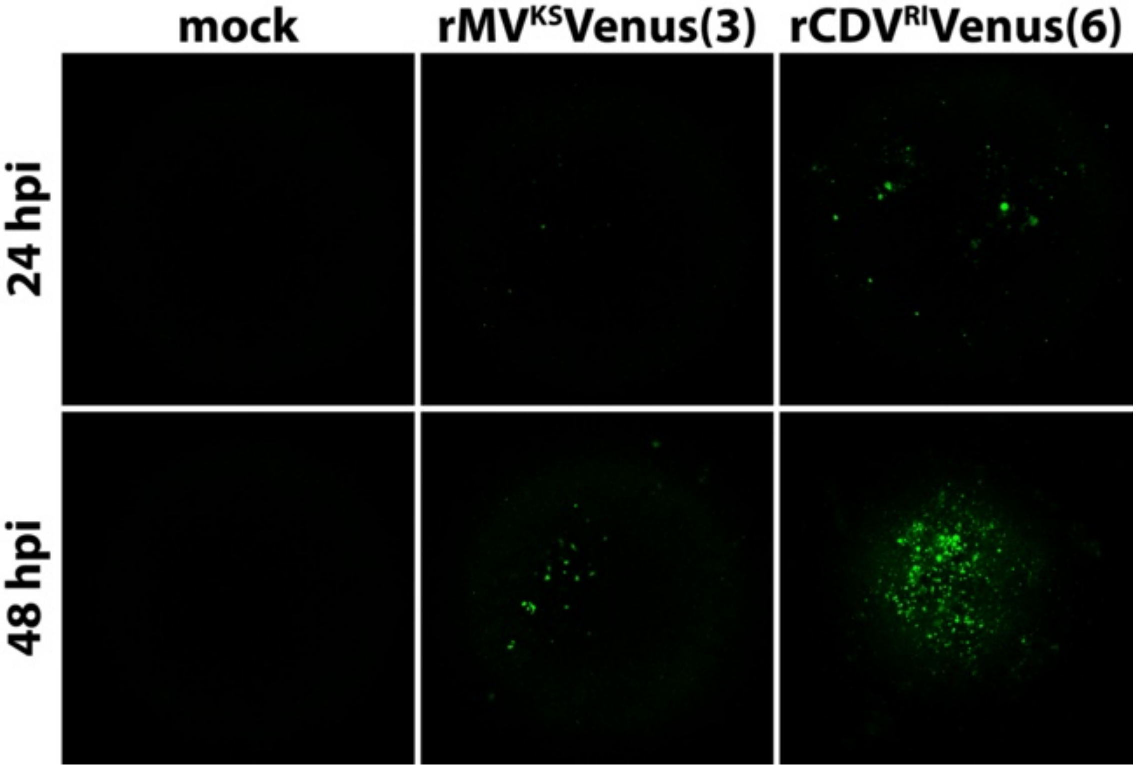

Figure S2. Ex vivo infection of raccoon WBC. (A) Confocal microscopy performed 24 and 48 hpi of raccoon WBC infected with mock, rMV^{KS}Venus(3) or rCDV^{RI}Venus(6). Quantification of infection percentages is shown in **Figure 1**.

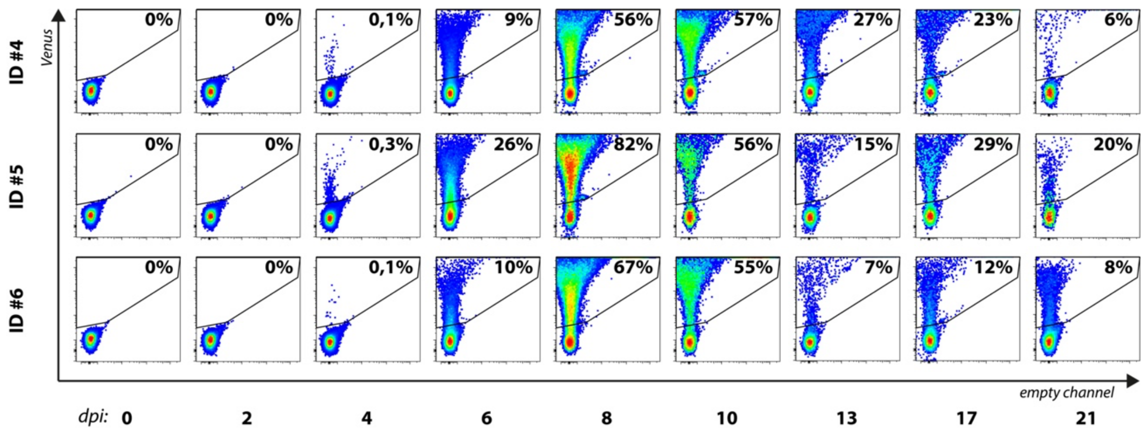

Figure S3. Detection of Venus-positive cells via flow cytometry. Venus-positive lymphocytes in peripheral blood were detected by flow cytometry. After gating the LIVE singlets lymphocytes (data not shown), the percentage of Venus-positive cells was determined by gating versus an empty fluorescent channel. Percentages Venus-positive cells detected at different time points in the animals sacrificed at 21 dpi are shown.

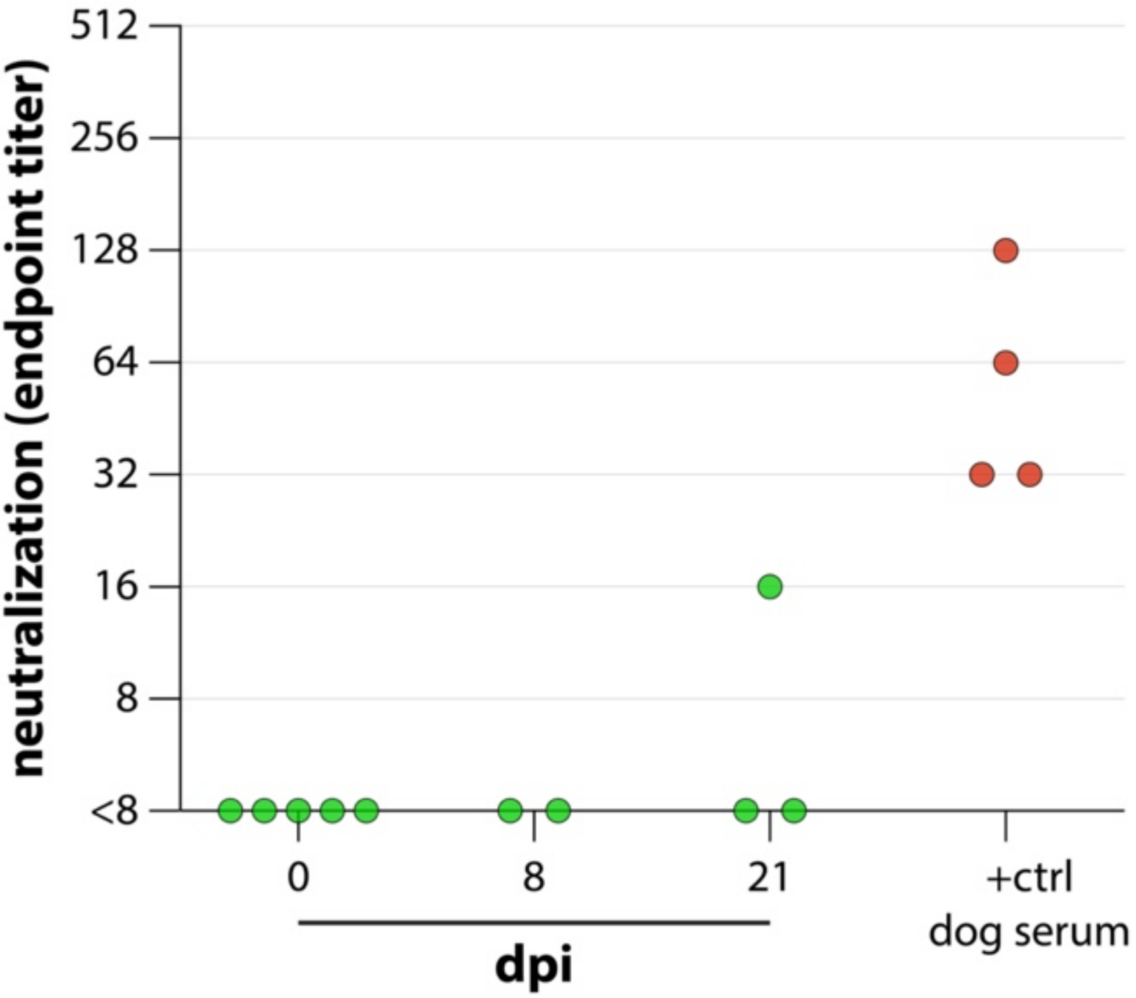

Figure S4. Neutralizing antibody titers in rCDV^{RI}Venus(6)-infected raccoons. Endpoint neutralization titers were determined by incubating serial serum dilutions with rCDV^{RI}Venus(6) and virus isolation on VDS cells. Samples obtained at 0, 6, 8 and 21 dpi were measured. Serum obtained from CDV-vaccinated dogs was included as positive control. Symbols represent individual animals.

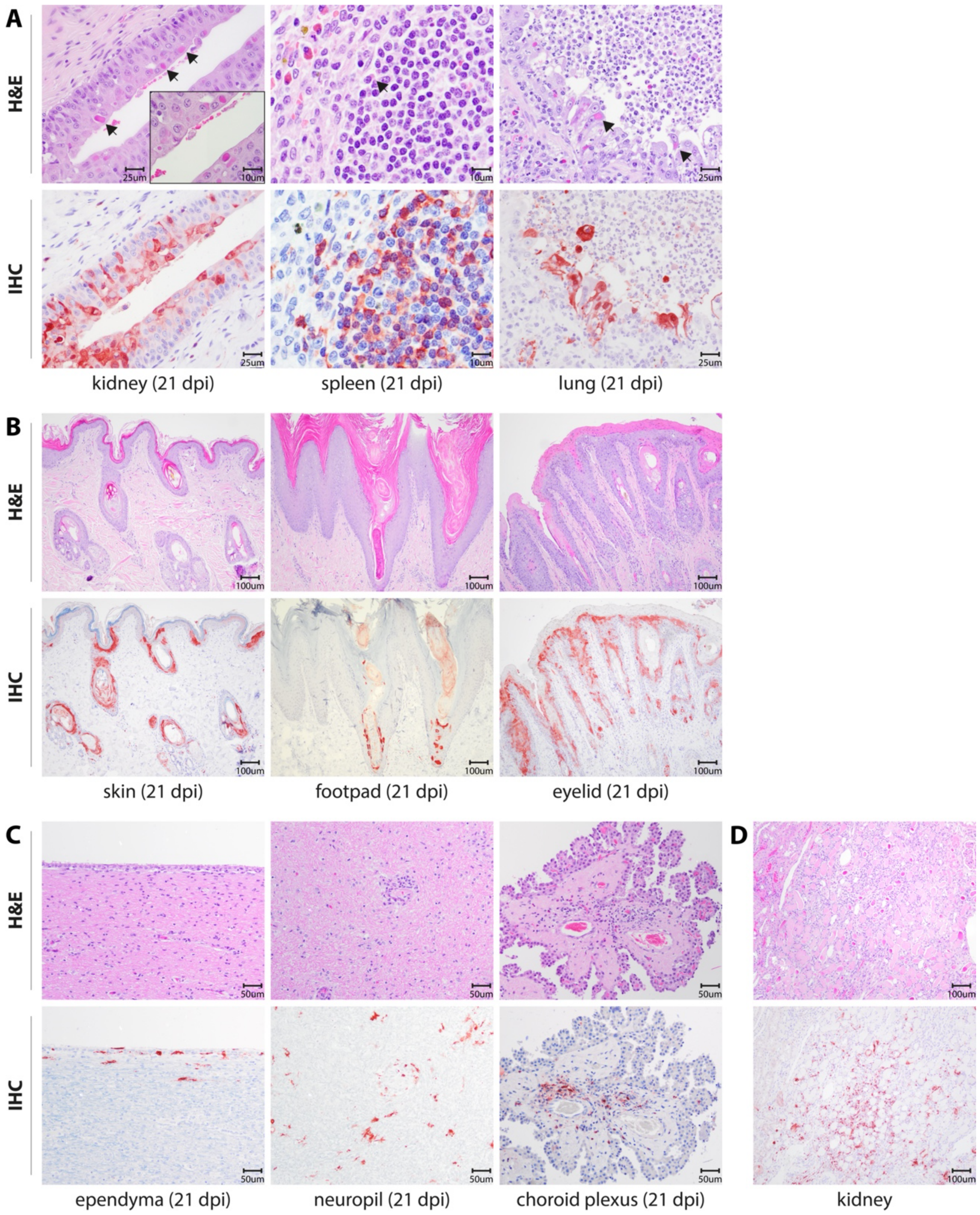

Figure S5. Histological and immunohistochemical evaluation of various tissues. (A) H&E staining of inclusion bodies in the kidney, spleen, and lung (all 21 dpi). Arrows indicate examples of inclusion bodies (top row). Antigen distribution by immunohistochemistry in the kidney, spleen, and lung (all 21 dpi) (bottom row). (B) H&E staining of evaluation of skin, footpad, and eyelid at 21 dpi (top row). Virus antigen distribution by immunohistochemistry (bottom row). (C) H&E staining of evaluation of the CNS (ependyma, neuropil, choroid plexus) (21 dpi) (top row). Virus antigen distribution by immunohistochemistry (bottom row). Infected cells include ependymal epithelial cells, astrocytes and axons. (D) H&E staining of Infarct with interstitial nephritis in the kidney (top row). Antigen distribution by Immunohistochemistry (bottom row).
